# Cadaveric Human Growth Hormone–Associated Creutzfeldt-Jakob Disease with Long Latency Period, United States

**DOI:** 10.3201/eid3106.241519

**Published:** 2025-06

**Authors:** Anatevka S. Ribeiro, Andrew B. Wolf, Ellen W. Leschek, Lawrence B. Schonberger, Joseph Y. Abrams, Ryan A. Maddox, Brian S. Appleby, Katie Glisic, Aaron Carlson, Elizabeth Matthews

**Affiliations:** University of California, Irvine, Orange, California, USA (A.S. Ribeiro); University of Colorado School of Medicine, Aurora, Colorado, USA (A.S. Ribeiro, A.B. Wolf); National Institutes of Health, National Institute of Diabetes, Digestive and Kidney Diseases, Bethesda, Maryland, USA (E.W. Leschek); Centers for Disease Control and Prevention, Atlanta, Georgia, USA (L.B. Schonberger, J.Y. Abrams, R.A. Maddox); Case Western Reserve University, National Prion Disease Pathology Surveillance Center, Cleveland, Ohio, USA (B.S Appleby, K. Glisic); University Hospitals Cleveland Medical Center, Cleveland (B.S. Appleby, K. Glisic); University of Colorado Anschutz Medical Campus, Aurora (A. Carlson, E. Matthews)

**Keywords:** Creutzfeldt-Jakob disease, CJD, prions and related diseases, sporadic, human growth hormone, myoclonus, PrP^Sc^ proteins, United States

## Abstract

We report a case of iatrogenic Creutzfeldt-Jakob disease (iCJD) after a 48.3-year incubation period in a patient treated with cadaveric human growth hormone. iCJD was pathologically confirmed; genetic analysis was negative for pathogenic mutations. Clinicians should consider iCJD in patients with progressive neurologic signs who had received cadaveric human growth hormone treatment.

Prion diseases are fatal neurodegenerative disorders caused by pathogenic misfolded prion protein (PrP^Sc^). PrP^Sc^ induces surrounding normal proteins to misfold, leading to a pathologic misfolding cascade that results in widespread neuronal cell death. Creutzfeldt-Jakob disease (CJD) is a prion disease that is considered transmissible because iatrogenic PrP^Sc^ exposure in specific circumstances can trigger the disease. 

The 3 forms of CJD are defined according to the mechanism by which PrP^Sc^ is acquired: sporadic (sCJD), iatrogenic (iCJD), and genetic (gCJD) (*1*,*2*). sCJD generally has a slower primarily cognitive nonfocal onset, but iCJD progresses rapidly with focal symptoms such as ataxia and jerky movements (*1*). iCJD has been a particular public health concern because of the potential for outbreaks. A well-described cause of iCJD is cadaveric human growth hormone (chGH), which was used to treat growth failure in ≈7,700 US patients during the 1960s through the 1980s through the National Hormone Pituitary Program (NHPP) (*3*). We report a case of iCJD after a long latency period in a patient treated with chGH in the 1970s.

## The Study

A 58-year-old woman was evaluated for a 2-week history of gait imbalance and tremors. Her medical history was notable for depression, cervical spine fusion, and idiopathic panhypopituitarism. She received chGH treatment for 9.3 years through NHPP, starting in late 1971 at 7 years of age.

Initial neurologic examination revealed frequent lateral movements of the head and trunk and hand movements that were irregular in amplitude and frequency without a null point that resolved with distraction. The examination was otherwise unremarkable, including gait evaluation, despite subjective gait impairment. The attending physician favored a diagnosis of functional neurologic disorder.

**Figure 1:**
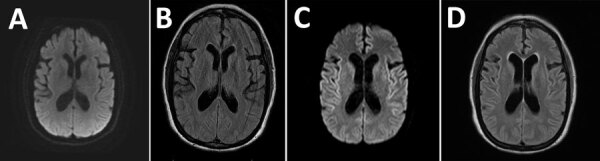
Magnetic resonance imaging of the brain from a case of cadaveric human growth hormone–associated Creutzfeldt-Jakob disease with long latency period, United States. A, B) Images obtained at initial clinical examination were unremarkable. C, D) Images obtained 3 months later demonstrated subtle areas of symmetric T2 hyperintensity in the insulae and frontotemporal lobes (C) and deep gray structures with diffusion restriction along the bilateral insulae and caudate heads without gadolinium enhancement (D).

Brain and cervical spine contrast magnetic resonance imaging (MRI) at initial examination was unremarkable (Figure 1, panels A, B). Mayo Clinic Autoimmune Movement Disorders Panel and HIV screening results were negative, and copper, vitamin E, and vitamin B12 levels were within reference ranges. The patient was referred to the movement disorders clinic and advised to start physical therapy and continue psychological treatment.

Over the next 4 weeks, urinary incontinence, worsening tremors, decreased speech output, and gait disturbance developed. She returned to the hospital with tachypnea, hyperekplexia, and appendicular rigidity. Her respiratory status and alertness rapidly declined, and she required intubation. Over several days, both stimulus-induced and spontaneous myoclonus developed, and she remained comatose. 

A repeat brain MRI 2 months after the initial MRI demonstrated subtle areas of symmetric T2 hyperintensity in the insulae and frontotemporal lobes and deep gray structures with diffusion restriction along the bilateral insulae and caudate heads without gadolinium enhancement (Figure 1, panels C, D). Continuous electroencephalogram showed frequent 1–2.5 Hz generalized periodic discharges lasting 3–9 seconds. Antinuclear antibody level was mildly positive at 1:160; all other serum test results, including autoantibody evaluations, were unremarkable. Cerebrospinal fluid viral and autoantibody results were negative. Oligoclonal bands were absent. Prion testing, performed at the National Prion Disease Pathology Surveillance Center (NPDPSC), Case Western Reserve University (Cleveland, Ohio), revealed elevated total τ of 9,104 pg/mL (reference <1,149 pg/mL) and elevated 14-3-3 protein level of 30,868 AU/mL (reference <1,999 AU/mL); real-time quaking-induced conversion assay was positive for prions. In accordance with her predetermined wishes, the patient was palliatively extubated and died. 

Autopsy performed at NPDPSC confirmed CJD by Western blot and immunohistochemistry analysis on brain tissue. *PRNP* genetic analysis was negative for pathogenic mutations, supporting a diagnosis of iCJD in the context of prior chGH treatment. The patient had a methionine/valine (M/V) polymorphism at codon 129 of the *PRNP* gene, which has been associated with longer latency periods in acquired human prion disease (*1*,*4*).

Cases of iCJD linked to chGH were first recognized in the United States in 1985 (*1*,*4*,*5*). Those reported iCJD cases led to the immediate cessation of chGH production and administration by the NHPP. Shortly thereafter, chGH was replaced with recombinant (biosynthetic) human growth hormone. Nevertheless, the iCJD outbreak linked to chGH continued because the latency period could be long among some previous chGH recipients. Latency or incubation periods for prion diseases have been shown to be variable and depend upon multiple factors, including the infectious particle dose, because higher doses are associated with shorter latency; route of infection, because central nervous system exposures are generally associated with shorter latency than peripheral exposures; and recipient genetics, particularly the codon 129 polymorphism in the *PRNP* gene (*6*). This patient was the 36th identified iCJD case among US NHPP chGH recipients and the 254th chGH-associated iCJD case reported worldwide as of January 2024 (*3*).

Determining the precise latency period for chGH-associated iCJD cases is often impossible because chGH exposure typically occurred over many years. Thus, latency must be estimated using one of several methods. First, latency can be calculated from the first dose of chGH to the onset of iCJD symptoms, which provides the maximum possible latency period. Second, latency can be estimated by calculating time from the midpoint of chGH treatment to iCJD symptom onset and is a standard estimate method used in other studies (*7*). Third, latency can be calculated as the time between the last dose of chGH and the onset of symptoms, which provides the minimum possible latency period. A final way to estimate latency is to use the midpoint of chGH treatment calculated from the first chGH dose to the end of 1977; then, latency is calculated between that midpoint and symptom onset. The reason for considering that method of calculation involves the epidemiology of the US outbreak, which suggests that the most likely source of iCJD infection came from chGH administered before or during 1977, even though most of the ≈7,700 NHPP recipients started chGH treatment after 1977 (*8*). Of note, none of the 36 US iCJD cases exclusively received post-1977 chGH, likely because the NHPP developed a new laboratory method to extract chGH from pituitary glands in 1977. That method included column purification to separate and collect multiple hormones from the glands, a procedure now known to have greatly reduced prion contamination (*3*,*8*). 

**Figure 2:**
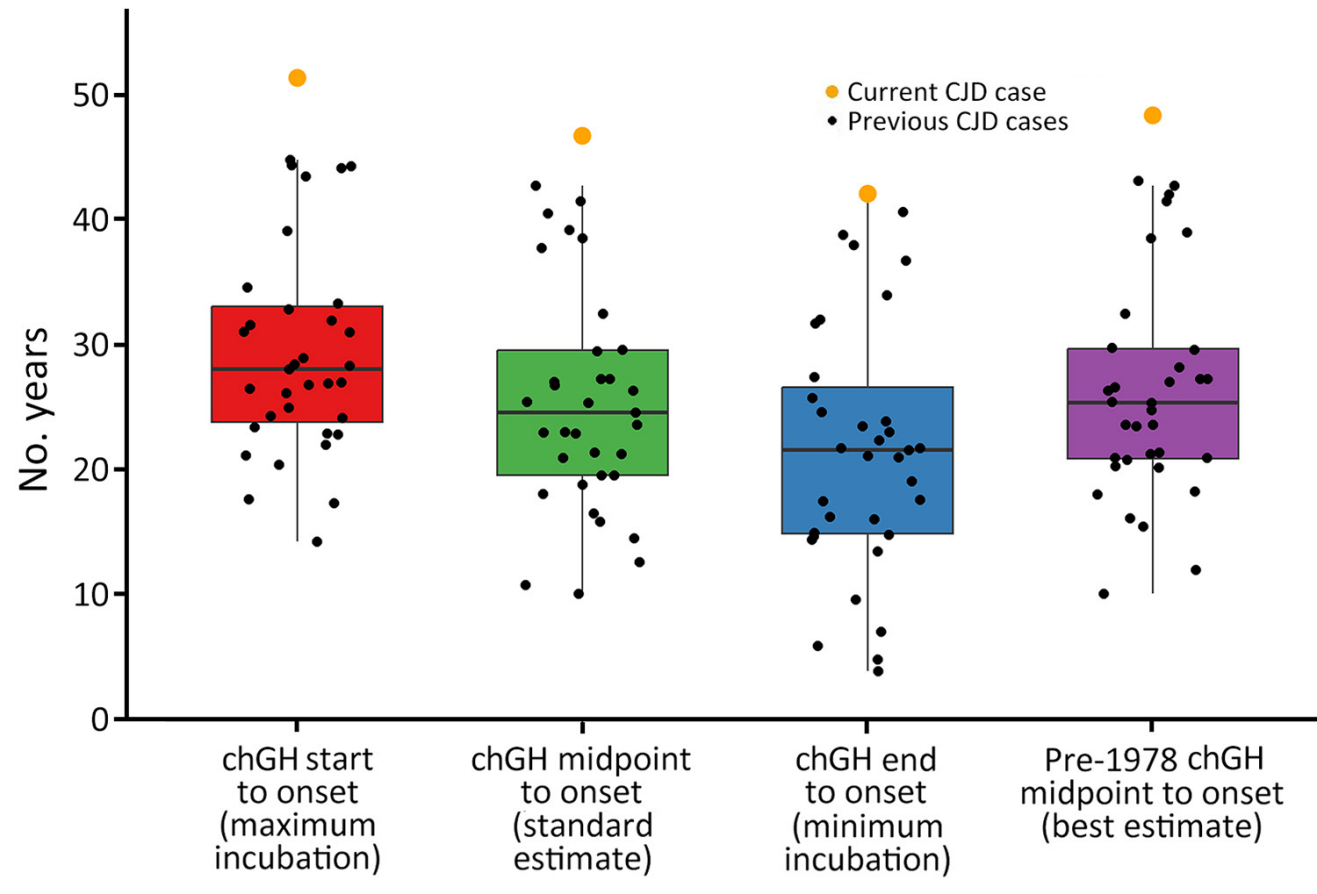
Comparison of latency estimates among US cases in a study of chGH-associated CJD with long latency period, United States. The patient in this report (current CJD case) was treated with chGH for 9.3 years starting in late 1971 at 7 years of age, and neurologic symptoms developed when she was 58 years of age. We used data from all 36 US cases of chGH-associated CJD to compare estimates of CJD latency periods via 4 methods: first dose of chGH to symptom onset (red), midpoint of chGH treatment to symptom onset (green), last dose of chGH to symptom (blue), and midpoint of pre-1978 chGH to symptom onset (purple). Using those 4 methods, we believe the last (purple box plot) to be the most accurate, giving a best estimate of 48.3 years for our patient’s latency period. Black dots indicate other CJD cases, box top and bottoms indicate 25th and 75th percentiles, horizontal lines inside boxes indicate medians, and whiskers extend to the furthest observed points within 1.5 times the interquartile range from the 25th and 75th percentiles. chGH, cadaveric human growth hormone; CJD, Creutzfeldt-Jakob disease.

We performed all 4 latency estimate calculations for our patient. First dose of chGH to symptom onset was 51.3 years, midpoint of chGH treatment to symptom onset was 46.7 years, last dose of chGH to symptom onset was 42.1 years, and midpoint of pre-1978 chGH to symptom onset was 48.3 years. We compared our patient’s latency estimate calculation to all US chGH-associated iCJD cases (Figure 2), and given the evidence implicating pre-1978 chGH, we considered the final method to provide the most accurate estimate of 48.3 years latency.

## Conclusions

In the ongoing US iCJD outbreak resulting from chGH, the shortest estimated latency was 10 years, whereas among the first 226 cases reported internationally, the shortest latency was 5 years, suggesting a lower level of prion contamination in the US NHPP-distributed chGH (*9*). In addition, experimental transmission studies in nonhuman primates using samples from all 76 lots of chGH retained on file at the US NHPP demonstrated that prion contamination was rare, random, and at a low level (*10*). That low level of contamination, the purification step introduced in the United States in 1977, and the peripheral route of administration created an environment that would be expected to result in longer latency periods. 

Although the US iCJD outbreak has slowed substantially, the potential for new cases remains, particularly among persons who are heterozygous M/V at codon 129 of the *PRNP* gene. Clinicians should recognize the continued possibility of chGH-associated CJD cases and include iCJD in the differential diagnosis for anyone with new neurologic symptoms and prior chGH exposure, particularly patients exposed to chGH before the 1977 updated purification process. 
